# Demographic and socioeconomic determinants of adherence in digital patient-reported outcomes among patients with chronic diseases

**DOI:** 10.1038/s41746-025-01899-2

**Published:** 2025-08-01

**Authors:** Janis Nikkhah, Alessandro Campione, Viktoria Steinbeck, Laura Wittich, Christoph Pross, Reinhard Busse

**Affiliations:** Department of Health Care Management, Technical University of Berlin, Berlin, Germany

**Keywords:** Outcomes research, Quality of life

## Abstract

Engaging patients in the digital collection of electronic patient-reported outcome measures (ePROMs) and experience measures (ePREMs) is desirable for equitable, patient-centred chronic disease management; however, adherence remain unclear. This study examined demographic and socioeconomic determinants of adherence using ePROMs and ePREMs collected from patients with asthma, chronic obstructive pulmonary disease, diabetes, and coronary artery disease across Germany. Of the 200,338 patients invited to complete digital surveys, 4657 consented (initiation; 2.32%) and 2375 completed at least one ePROM (implementation; 51.00% of initiation). Initiation was highest among asthma patients (3.42%) and lowest among those aged ≥75 years (1.09%). Implementation followed an inverse U-shaped age pattern and was lowest among patients with diabetes and those with low or unreported income. Findings indicate barriers to adherence associated with demographic and socioeconomic factors. Strategies such as inclusive engagement, integration of surveys into clinical care, and clinical endorsement may improve adherence. Trial registration: drks.de; Identifier: DRKS00031656.

## Introduction

Traditionally, healthcare quality has been assessed based on clinical indicators, such as clinician-reported symptom improvement and hospitalization rates^1^. An analysis of clinical guidelines demonstrated a predominant focus on process quality among quality indicators, with 86% in Germany and 40% in the United Kingdom (UK) assessing processes, while only 8% in Germany and 21% in the UK measured outcome quality^2^. These findings underscore the necessity of a more balanced approach to healthcare quality assessment, emphasizing the patient perspective. In this context, patient-reported outcome (PROMs) and experience measures (PREMs) include validated questionnaires that offer the potential to provide a more comprehensive evaluation of healthcare quality from the patient’s perspective. Their adoption is expanding across healthcare providers, medical conditions, and countries, offering valuable insights into patients’ perceived health status, treatment effectiveness, and overall satisfaction with care^3–5^.

The urgency of addressing chronic diseases is evident as their global burden and associated care costs continue to rise^6–8^. Healthcare systems face substantial challenges due to the growing prevalence of chronic diseases and long-term demands of chronic care^9,10^. PROMs and PREMs enable tailored healthcare services to better meet patients’ needs as shown in other specialties such as orthopaedics and oncology, with evidence for benefits for patients with chronic disease slowly emerging^11–15^. Digital collection is expanding, enabling large-scale, cost-efficient data collection and analysis^16,17^. In addition, digital platforms can facilitate transparency and allow for meaningful comparisons of healthcare across providers and international settings^18–20^. At the same time, the perceived utility of digital health tools among clinicians may vary depending on the clinical context, and their meaningful integration into routine care requires alignment with existing workflows^21,22^. In Germany, the hospital reform expert commission, established in 2022 and advising the Ministry of Health, emphasizes the potential of digital PROM (ePROM) and PREM (ePREM) collection to strengthen healthcare quality assessment and transparency^23^.

Previous studies have examined adherence determinants across various disease populations^24^, revealing disparities by age^25,26^, gender^27,28^, healthcare utilization patterns^27,29,30^, and educational background^25^. Despite these insights, a research gap remains regarding the patient characteristics associated with adherence in ePROMs and ePREM collection among patients with chronic diseases. Addressing this gap is essential to ensure that findings derived from the participating cohorts are generalizable to the broader target population and thereby inform patient-centred quality assessments^26^.

This study investigates the association between demographic and socioeconomic characteristics and survey adherence among patients with chronic diseases across Germany, considering three levels of adherence^31^: (1) Who consents to participate in the first place (initiation)? (2) Who completes at least one questionnaire when prior account registration and validation are required (implementation)? (3) Who remains engaged and regularly responds to ePROM and ePREM surveys over time (persistence)? This differentiated approach ensures a detailed understanding of adherence patterns that can inform future digital health survey designs to enhance the representativeness of collected data for the broader chronic patient population.

## Results

### Patient characteristics

**Fig. 1:**
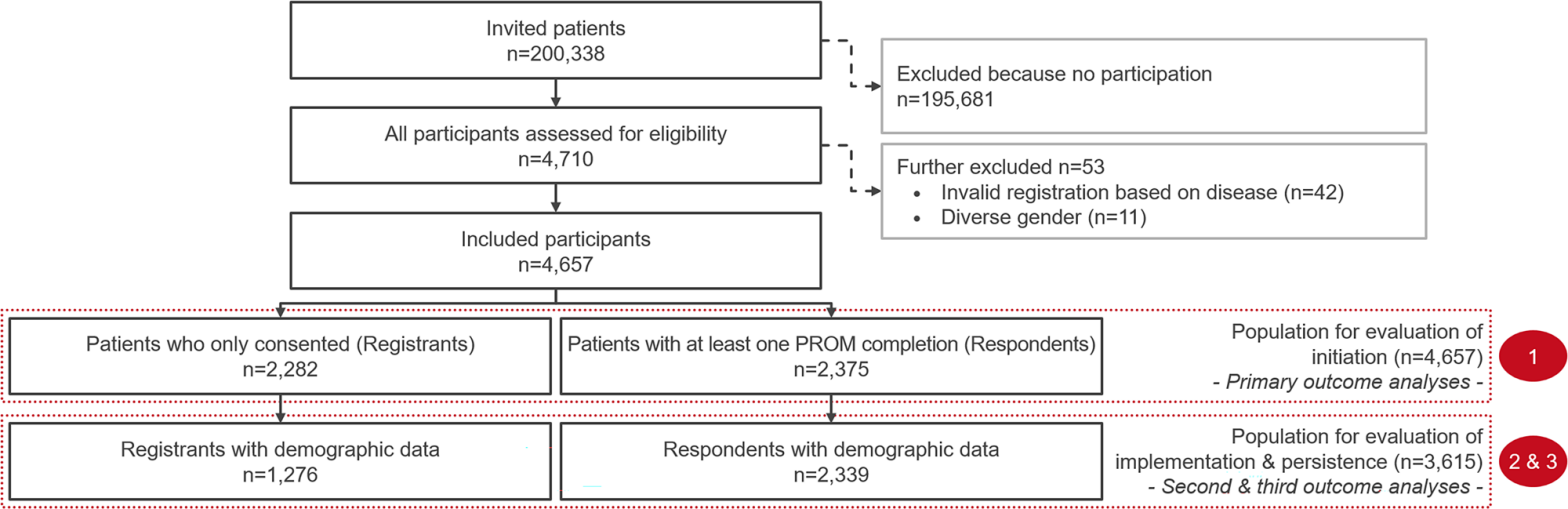
STROBE-flowchart of patient inclusion. Depicts the flow chart of patients invited to the study (*n* = 200,338) and reasons for inclusion. Patients who did not participate (*n* = 195,681) were excluded. Of the 4710 initial participants, 42 were excluded due to invalid registration based on disease criteria, and 11 participants identifying as diverse gender were excluded due to low volumes. The final study cohort comprised 4657 participants (initiated), including 2282 who only consented but did not complete ePROMs (registrants), and 2375 who completed account registration and at least one ePROM survey (respondents; implemented).

Of the 200,338 patients with chronic diseases invited to the PROMchronic study, 4657 (2.32% initiation) participated. Of these, 2282 (1.14%) only completed study consent (registrants), while 2375 (1.19%; 51.00% of initiation) completed at least one ePROM survey (respondents), see Fig. 1. Due to the small sample size, participants who identified as gender-diverse (*n* = 11) were excluded. The respondents included 930 patients with asthma, 501 with CAD, 499 with COPD, and 445 with type 1 or type 2 diabetes. Further details on the demographics and socioeconomic characteristics of the study population are presented in Table 1.

**Table 1:** Descriptives of patient characteristics and responses

	Participants (with initiation)				Non-participants (of total invited)	Total invited
	Total (of total invited)	Registrants; only initiated (of participants)	Respondents (with implementation)			
			(of participants; initiated)	Responses (avg. per respondent)		
Total	**4657 (2.32%)**	*2282 (49.00%)*	*2375 (51.00%)*	5745 (2.42)	**195,681 (97.68%)**	**200,388 (100%)**
Asthma	**1718 (3.42%)**	*788 (45.87%)*	*930 (54.13%)*	2275 (2.45)	**48,506 (96.58%)**	**50,224 (100%)**
COPD	**1004 (2.01%)**	*505 (50.30%)*	*499 (49.70%)*	1200 (2.40)	**48,996 (97.99%)**	**50,000 (100%)**
Diabetes	**949 (1.89%)**	*504 (53.11%)*	*445 (46.89%)*	1076 (2.42)	**49,165 (98.11%)**	**50,114 (100%)**
CAD	**986 (1.97%)**	*485 (49.19%)*	*501 (50.81%)*	1194 (2.38)	**49,014 (98.03%)**	**50,000 (100%)**
Male	**2105 (2.37%)**	*1035 (49.17%)*	*1070 (50.83%)*	2546 (2.38)	**86,386 (97.62%)**	**88,491 (100%)**
Female	**2552 (2.28%)**	*1247 (48.86%)*	*1305 (51.14%)*	3199 (2.45)	**109,295 (97.72%)**	**111,847 (100%)**
18–44 years	**500 (3.47%)**	*248 (49.60%)*	*252 (50.40%)*	578 (2.29)	**13,898 (96.53%)**	**14,398 (100%)**
45–64 years	**2110 (3.42%)**	*937 (44.41%)*	*1173 (55.59%)*	2884 (2.46)	**59,600 (96.58%)**	**61,710 (100%)**
65–74 years	**1311 (2.31%)**	*650 (49.58%)*	*661 (50.42%)*	1579 (2.39)	**55,399 (97.69%)**	**56,710 (100%)**
≥75 years	**736 (1.09%)**	*447 (60.73%)*	*289 (39.27%)*	704 (2.44)	**66,787 (98.91%)**	**67,520 (100%)**
**Participants’ demographic and socioeconomic data as available (*n*** **=** **3615 out of 4657 total participants, for 36 respondents who gave 51 responses demographic and socioeconomic data was not available)**						
Demographic and socioeconomic data	***n*** **=** **3,615 (100%)**	*n* = 1,276 (35.30%)	*n* = 2,339 (64.70%)	*n* = 5,694 (2.43)		
Age, mean [SD]	**61.655 [13.097]**	*62.727 [13.633]*	*60.625 [12.476]*			
Lower education	**643 (100%)**	*263 (40.90%)*	*380 (59.10%)*	922 (2.43)		
Middle education	**1196 (100%)**	*406 (33.95%)*	*790 (66.05%)*	1902 (2.41)		
Higher education	**1700 (100%)**	*574 (33.76%)*	*1126 (65.24%)*	2778 (2.47)		
Other education	**76 (100%)**	*33 (42.42%)*	*43 (56.58%)*	92 (2.14)		
Lower income	**668 (100%)**	*241 (36.08%)*	*427 (63.92%)*	1025 (2.40)		
Middle income	**1011 (100%)**	*348 (34.42%)*	*663 (65.58%)*	1633 (2.46)		
Higher income	**877 (100%)**	*245 (27.94%)*	*632 (72.06%)*	1530 (2.42)		
Income N/A	**1059 (100%)**	*442 (41.74%)*	*617 (58.26%)*	1506 (2.44)		

Bold values indicate the primary comparison groups in the analysis: participants versus non-participants. Italicized values represent nested subgroups within the participant population: registrants versus respondents.

The highest initiation and implementation were observed among asthma patients, with initiation (3.42%) and implementation (54.13% of initiation) above those for other diseases, which ranged between 1.97–2.01% for initiation and 46.89–50.81% for implementation. While women comprised the majority of the study population (55.83%), the initiation was slightly higher for men (2.37% male vs. 2.28% female). The implementation was similar across gender with 51.14% female vs. 50.83% male participants. While patients aged over 75 years represented the largest share of the invited population (33.70%), the highest initiation was observed in the 18–44 (3.47%) and 45–65-year (3.42%) age groups. Initiation and implementation were lowest in patients aged over 75 with 1.09% and 39.27%, respectively.

**Fig. 2:**
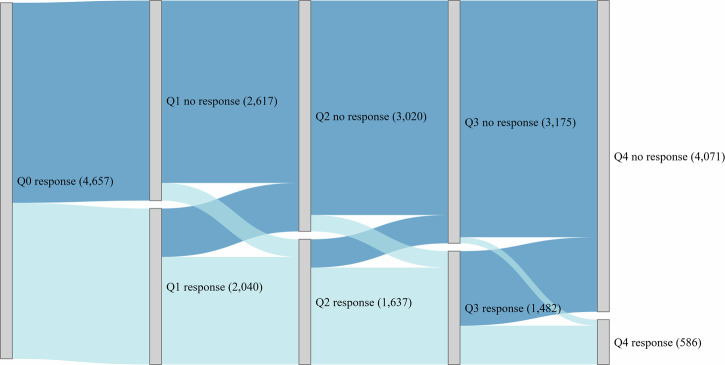
Implementation and persistence over time. Sankey plot illustrating the segmentation of study participants based on their adherence status. The first level includes all participants. Among participants, two groups emerged: Light blue represents respondents, while dark blue represents non-respondents. The width of the flows reflects the number of participants transitioning between response and non-response over time.

Figure 2 illustrates the implementation and persistence across survey periods. In total this study considered 5745 ePROM responses. Among the 4657 participants (Q0 response), 2040 completed the first-quarter ePROM (Q1 response), while 2617 did not respond (Q1 no response). In the second quarter, 1637 responded (Q2 response), with 3020 non-responders (Q2 no response). The decline continued in the third quarter, with 1482 responses (Q3 response) and 3175 non-responses (Q3 no response). A stronger decline was observed in the fourth quarter, where responses dropped to 586 (Q4 response), while 4071 did not respond (Q4 no response).

### Model comparison of demographic drivers for initiation (1)

The model comparison results (Table 2) indicated that model 5, a simplified model using a cloglog link function, performed best. It achieved the lowest AIC, indicating the best balance between model fit and complexity while maintaining low deviance, a high percentage of deviance explained, high AUC, and the lowest Brier’s score, which measures prediction accuracy. Full details of the model comparison are provided in Supplementary Table 1.

**Table 2:** Result of model comparison to determine predictors of initiation

	Model 1 Full-Logit	Model 2 Simple-Logit	Model 3 Main-Logit	Model 4 Full-Cloglog	Model 5 Simple-Cloglog	Model 6 Main-Cloglog
AIC	42,991.58	42,987.18	43,193.50	42,991.55	**42,987.14**	43,193.79
Dev.	42,945.58	42,947.18	43,177.50	42,945.55	**42,947.14**	43,177.79
Dev. explained	2.93%	2.92%	2.40%	2.93%	**2.92%**	2.40%
AUC	0.64791	0.64780	0.63626	0.64791	**0.64780**	0.63626
Brier’s Score	0.02256	0.02256	0.02259	0.02256	**0.02256**	0.02259
Pseudo *R*^2^	0.03	0.03	0.02	0.03	**0.03**	0.02

Bold values indicate the best performing model 5 (simple-cloglog).

### Predictors of initiation (1)

The evaluation of the relative predictor contributions to study initiation was conducted using the best performing model 5. Increasing age was generally associated with lower initiation, with significant negative association observed for the 65–74 years (β = -0.502, *p* < 0.001) and ≥75 years (β = -1.408, *p* < 0.001) age groups compared to the reference category (18–44 years). Gender was a significant predictor in interaction models. While male gender showed a negative association with initiation (β = -0.597, *p* < 0.001), this was mitigated by a significant interaction between age and gender. For instance, men aged ≥75 years were significantly more likely to initiate than their respective reference categories (β = 1.381, *p* < 0.001). Overall, disease effects were not significant, but interactions between age groups and disease revealed varying associations. Patients aged 45–64 years with diabetes had a significantly reduced likelihood of initiation (β = -0.510, *p* < 0.001), with a stronger negative association for those aged 65–74 years (β = -0.644, *p* < 0.001) and ≥75 years (β = -0.824, *p* < 0.001). No significant interaction effects were observed for other chronic diseases.

Average marginal effects (AME) (Table 3) further quantified predictor associations. The estimated initiation probability for the reference group (asthma, female, 18–44 years) was 4.44%, closely aligning with the observed raw percentage (4.58%). Compared to asthma patients, those with CAD were less likely (-0.69 percentage points (pp); SE: 0.14 pp; *p* < 0.001) to initiate, while initiation was also lower for COPD (-0.95 pp; SE: 0.11; *p* < 0.001) and diabetes (-1.08 pp; SE: 0.11 pp; *p* < 0.001). Gender associations showed a 0.18 pp increase in initiation for men (SE: 0.07 pp; *p* = 0.014). Age-group associations remained strong; patients within the age-group 65–74 years had significantly lower probability of initiation (-0.93 pp; SE: 0.35 pp; *p* = 0.004), with a larger association for those aged ≥75 years (-2.14 pp; SE: 0.35 pp; *p* < 0.001).

**Table 3:** Average marginal effects of initiation predictors in best performing model (model 5)

	AME	SE	z	p	CI (lower | upper)
Asthma	Reference group				
COPD	−0.0095	0.0011	−8.4469	<0.001	−0.0117 | −0.0073
Diabetes	−0.0108	0.0011	−10.2147	<0.001	−0.0129 | −0.0087
CAD	−0.0069	0.0014	−4.7915	<0.001	−0.0098 | −0.0041
Female	Reference group				
Male	0.0018	0.0007	2.4534	0.0142	0.0004 | 0.0032
18–44 years	Reference group				
45–64 years	0.0002	0.0035	0.0443	0.9648	−0.0067 | 0.0070
65–74 years	−0.0093	0.0035	−2.6931	0.0071	−0.0161 | −0.0025
≥75 years	−0.0214	0.0034	−6.2460	<0.001	−0.0281 | −0.0147

### Predictors of implementation (2) and persistence (3)

Separate hurdle regression models were developed to predict implementation and persistence among participants with available demographic and socioeconomic data (*n* = 3,615, see Fig. 2). The Box-Tidwell test for the age variable was significant, indicating non-linearity in log-odds, therefore squared age was included in the model (Maximum likelihood estimate of lambda = 7.9703, *p* < 0.001). No variables exhibited problematic VIF, hence, no variables required exclusion. The results of the hurdle regression model are presented in Table 4.

**Table 4:** Hurdle regression on ePROM completions—implementation (binary component) and persistence (count component)

	Model 1: Implementation		Hurdle overcome →	Model 2: Persistence	
	β	*p*-value		β	*p*-value
Intercept	1.126	<0.001		0.877	<0.001
Female	Reference group			Reference group	
Male	−0.055	0.474		−0.005	0.257
Age	0.015	<0.001		0.002	0.213
Age-squared	−0.001	<0.001			
Asthma	Reference group			Reference group	
COPD	−0.154	0.131		−0.046	0.426
Diabetes	−0.311	0.002		−0.023	0.695
CAD	0.021	0.849		−0.085	0.165
Higher education	Reference group				
Lower education	−0.179	0.072			
Middle education	−0.002	0.985			
Other education	−0.310	0.198			
Higher income	Reference group				
Lower income	−0.268	0.019			
Middle income	−0.221	0.031			
N/A income	−0.549	<0.001			
AIC	4628.938			6731.428	
BIC	4709.445			6771.731	
Pseudo R^2^	0.034				

Model 1: Binary component—logistic regression; logit link. Model 2: Count component—truncated generalized Poisson; log-link.

### Overcoming the hurdle—implementation (2)

**Fig. 3:**
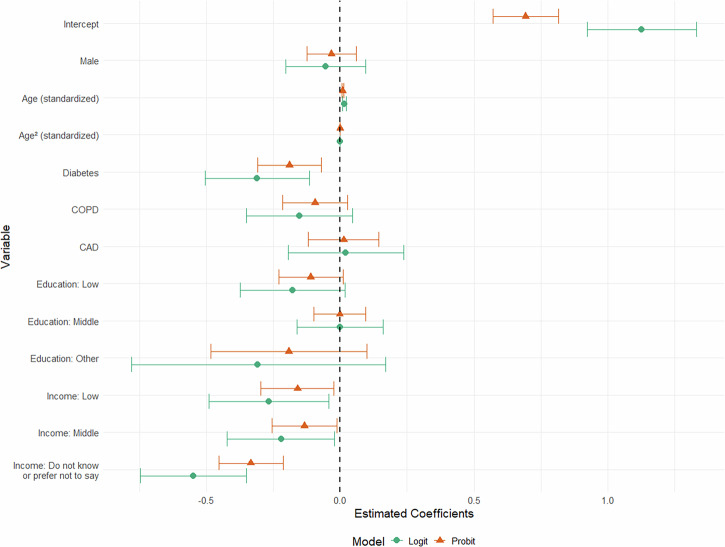
Coefficient plot comparing logit and probit models on implementation—binary component. Coefficient plot with 95% confidence interval from the implementation component of the hurdle regression model (logit and probit), with overcoming the hurdle as outcome, and female reference group at median age with asthma, higher education and higher income. There was a negative association with diabetes disease and lower or not specified income group. Age was positively associated with implementation whereas negative association of age-squared revealed that the association followed an inverse U-shaped curve with lower probability at younger and older age. COPD chronic obstructive pulmonary disease, CAD coronary artery disease.

For the implementation analyses, a logit link function provided the best model performance with the lowest AIC. Coefficient results from the separate component models are presented in Fig. 3 and AMEs of the logit model are presented in Table 5. The intercept revealed the estimated average probability of the reference group (female of median age with asthma, higher education, and higher income) to overcome the hurdle of first ePROM completion at 75.52% (*p* < 0.001). The implementation was significantly lower for registered diabetes patients (AME: -7.06 pp; SE: 2.27 pp; *p* = 0.002). Additionally, significant socioeconomic factors included lower household income (AME: -5.75 pp; SE: 2.47 pp; *p* = 0.020) and middle household income (AME: -4.72 pp; SE: 2.17 pp; *p* = 0.030), leading to a lower implementation. The lowest implementation occurred among participants who did not know or preferred not to disclose their household income (AME: -12.29 pp; SE: 2.22 pp; *p* < 0.001). The AME for age (0.34 pp; SE: 0.08 pp; p < 0.001) indicated a higher implementation with increasing age. This was contradicted by the negative AME for age-squared (-0.01 pp; SE: 0.00 pp; *p* < 0.001), revealing an inverse U-shaped relationship, where implementation peaked around 51.6 years before declining. Other coefficients did not show significant associations on overcoming the hurdle.

**Table 5:** Average marginal effects of predictors of overcoming the hurdle of account registration and ePROM completion—implementation

	AME	SE	z	p	CI (lower | upper)
Age	0.0034	0.0008	4.4403	<0.001	0.0019 | 0.0049
Age-squared	−0.0001	0.0000	−4.3138	<0.001	−0.0002 | −0.0001
Asthma	Reference group				
COPD	−0.0341	0.0227	−1.5051	0.132	−0.0785 | 0.0103
Diabetes	−0.0706	0.0227	−3.1105	0.002	−0.1151 | −0.0261
CAD	0.0046	0.0239	0.1908	0.849	−0.0422 | 0.0513
Female	Reference group				
Male	−0.0122	0.0170	−0.7147	0.475	−0.0456 | 0.0212
Higher education	Reference group				
Lower education	−0.0406	0.0228	−1.7808	0.075	−0.0852 | 0.0041
Middle education	−0.0003	0.0181	−0.0188	0.985	−0.0357 | 0.0351
Other education	−0.0712	0.0570	−1.2490	0.212	−0.1830 | 0.0406
Higher income	Reference group				
Lower income	−0.0575	0.0247	−2.3312	0.020	−0.1058 | −0.0092
Middle income	−0.0472	0.0217	−2.1735	0.030	−0.0897 | −0.0046
N/A income	−0.1229	0.0222	−5.5448	<0.001	−0.1663 | −0.0794

### Persistence (3)

For persistence, a truncated generalized Poisson model with log-link performed best across specifications (see Supplementary Table 2 for full model comparison). Once the hurdle was overcome, the persistence model did not identify any significant predictors for the number of responses in any of the tested specifications. Although not statistically significant, the model estimated an average of 2.40 responses for a reference patient (female of median age with asthma). Age was not a significant predictor, but it exhibited a similar trend to the implementation, with a positive association of each year over the median and a negative association of squared median-centred age, suggesting a diminishing association at older ages. Compared to asthma respondents, respondents with one of the other diseases had lower persistence, as did male patients. A standard Poisson reference model indicated underdispersion in the persistence (dispersion ratio: 0.468). To address this, the adapted truncated generalized Poisson model estimated a dispersion parameter of $\delta =0.511$, indicating moderate underdispersion. The intercept of the persistence was the only consistent statistically significant parameter and represented the estimated average response number for a female respondent of median age with asthma (beta = 0.877 (0.018), *p* < 0.001, exp(0.877) = 2.40 responses on average, c.p.) after overcoming the hurdle.

## Discussion

This study examined demographic and socioeconomic factors associated with adherence in a nationwide digital health survey incorporating ePROMs and ePREMs among patients with chronic diseases in Germany. Despite following the latest recommendations for digital health survey design^32–34^, overall adherence was low and declined over time, underscoring challenges in initiation, implementation, and persistence^35,36^. To minimize respondent burden, the survey was designed to take approximately 10 min to complete, and two email reminders were sent for each open survey task^33^. Further incentives such as prize draws or a cash or non-cash incentive were not permissible due to regulatory and ethical constraints. Additional factors potentially contributing to low adherence include survey fatigue, particularly following increased survey activity during and after the COVID-19 pandemic, as observed in other medical fields^37^. Moreover, initial contact through the health insurance provider may have triggered concerns about data privacy, reducing willingness to participate.

Initiation was lower among patients aged 65 to 74 years and those aged 75 years or older. This finding is consistent with previous research^38,39^, though the association in this study was more pronounced among women. It is noteworthy that while male sex overall showed a negative association with initiation, this was mitigated by a significant interaction with age. Specifically, male patients aged 75 years or older were more likely to initiate than would be expected based on the main effects of age and gender alone. One possible explanation is higher digital literacy among employed compared to non-employed individuals, combined with a higher labour market participation among men in older cohorts in Germany^40^. Diabetes was the only disease linked to lower initiation among middle-aged and older adults. This aligns with a prior study reporting a decrease in participation beyond the age of 44 years^41^. More generally, other important reasons for low initiation may include the absence of physician or other healthcare professional involvement, which has been identified as a key facilitator in PROM adherence^42,43^. In the PROMchronic trial, recruiting was initiated via invitation letter from the patients’ health insurance provider without further contact to or endorsement of physicians or other healthcare professionals. In contrast, studies involving patient populations undergoing acute treatment, such as e.g. cancer care^44,45^ or surgical interventions^46^, have reported notably higher adherence, particularly at the initiation stage. This may point to the importance of contact with healthcare professionals in motivating patient participation. Additionally, previous research suggests that the utilization of alert systems, such as automated emails or telephone contacts at certain PROM score thresholds, can improve overall adherence of patients in ePROM surveys, as patients perceive value in being remotely monitored^47^. Furthermore, other studies’ findings suggest that initiation tend to be lower in fully digital collection methods^48,49^, however, this effect may be partially mitigated through regular reminder mechanisms^48,50^.

The mandatory account registration process, including password generation and verification, required by German data privacy regulations, may have posed a significant barrier to adherence, as reflected in the low implementation. As observed in another study, age followed an inverse U-shaped association with implementation. The likelihood of overcoming the hurdle of account registration and first ePROM completion increased up to the age of 51.6 years and declined thereafter^51^. A study by Berg et al. (2024) involving 13 countries reported comparable results for four countries, while in five countries the turning point occurred earlier, and in the remaining countries participation steadily declined with increasing age. Additionally, the results of this study indicated that lower income and non-disclosure of income were associated with reduced implementation^52^. However, once the initial hurdle of implementation was overcome, demographic or socioeconomic characteristics were not significantly associated with the persistence. Still, older age^41,53^, female sex^41,53^, and asthma disease were linked to slightly higher persistence compared to reference groups.

The persistence model did not yield any significant associations with the variables available for analyses. One possible explanation could include that patients who responded at least once may have constituted a relatively homogenous group with respect to unobserved variables, such as strong intrinsic willingness or curiosity to participate in studies. These unobserved characteristics could have helped them overcome the processual hurdle of account registration, leaving limited variation to explain differences for the later persistence.

Analyses were conducted on complete cases only, as some observations for demographic or socioeconomic variables were missing (*n* = 36) or responded with “do not know or prefer not to answer” (*n* = 617). Although the overall rate of missing data was low, the non-specific response category may have included patients whose inclusion could have improved the power to detect associations. This limitation is particularly relevant to the willingness to disclose household income, which was suspected to be non-random.

This study has several strengths. It is the first study to investigate the utilization of ePROMs among patients with chronic diseases in Germany. While the parallel OECD PaRIS study is assessing primary care through the utilization of digital surveys internationally, it does not include Germany, which represents the largest European healthcare market and has a high prevalence of chronic conditions^54^. Unlike the OECD’s provider-based recruitment approach^54,55^, the PROMchronic employed a centralized strategy independent of specific care providers. This may reduce provider-driven selection bias and yield results that are more representative of the broader patient population. The PROMchronic study and the present analyses provide important insights into how Germany can collect ePROMs and ePREMs for future international comparisons, and which aspects must be addressed to gather information of a representative cohort. A key strength of the study lies in its large initial recruitment sample. Moreover, the inclusion of multiple chronic disease groups enabled condition-specific comparisons and supported more targeted recommendations. Another strength was the flexible digital access: patients received both a link and a QR code in the invitation letter and were able to switch between devices across survey tasks.

Several limitations should also be acknowledged. Adherence declined over the course of the study period. Although PROMs validated in German patient cohorts were utilized and feedback by patient organisations was obtained on the selected questionnaires, the limited involvement of patients in the initial study design may have contributed to a low perceived relevance, as well as the low implementation and persistence observed. Future research projects should consider co-development with end users, e.g., by patient and public involvement (PPI) from the very beginning of the study^56^, to ensure content relevance and enhance the perceived value of participation and engagement. Patients identifying as gender-diverse were excluded due to the small sample size. The study included only a limited set of demographic variables, which were available only in aggregated form, and no socioeconomic information was available for non-participants. Other unmeasured factors such as digital literacy, access to technology or health literacy, may have affected the adherence. Another limitation of the study is the exclusion of patients enrolled in multiple DMPs. While this approach allowed for clear linkage of patients to a single chronic disease, it may have reduced the generalizability of findings. Multimorbidity is highly prevalent among patients with chronic diseases^57^, and excluding patients enrolled in multiple DMPs likely resulted in their underrepresentation. Furthermore, the study did not include more detailed, disease-specific characteristics that may have influenced adherence. This could help explain the observed significance of interaction between age and disease, particularly diabetes, despite the overall effects of disease being non-significant.

This study provides new insights into the utilization of digital health surveys incorporating ePROMs and ePREMs to assess care quality among patients with chronic diseases. The findings reveal that patient demographics are significantly associated with adherence, with asthma patients showing the highest engagement, whereas diabetes patients and older age groups exhibited the lowest implementation and persistence. The results also underscore that overcoming the hurdle of account registration and completing at least one ePROM survey is a crucial barrier for adherence, particularly for older patients and those with lower income. The inability to simplify the sign-in process during the study design phase was partly due to regulatory requirements of the ePROM tool, which limited the implementation of patient-centred onboarding adaptations. Additionally, the observed relationship between age and implementation revealed reduced adherence among both younger and older patients, suggesting that different engagement strategies may be needed for different age groups. Therefore, the findings suggest that minimizing initiation and implementation barriers and simplifying the setup process is essential to encourage broader and more inclusive participation and improve adherence in digital health surveys. Future research should explore alternative methods for collecting ePROM and ePREM data within clinical care and reinforce mechanisms for adherence in patients with chronic diseases. Addressing these challenges is crucial for the routine integration of ePROMs and ePREMs into chronic disease care and their secondary use for quality assessments.

## Methods

### Study design and population

Based on the observational, prospective cohort study PROMchronic, this paper aims to examine the adherence of patients with chronic diseases in ePROM and ePREM surveys, evaluate the overall initiation and implementation, and analyse temporal variations in persistence across patient subgroups stratified by disease, age, income and gender over time. The design of the PROMchronic study has been previously described in the PROMchronic study protocol^58^.

Patients diagnosed with one of the following chronic diseases – asthma, diabetes (type 1 or 2), chronic obstructive pulmonary disease (COPD), or coronary artery disease (CAD) – and insured through the sickness fund BARMER, one of Germany’s largest health insurance providers covering over 10% of the population^59^, were recruited nationwide for the study. Using a randomized, stratified selection process, a total of 200,338 patients (around 50,000 per disease) received an invitation letter from their statutory health insurance provider in October 2023. Patients were required to be at least 18 years old with a confirmed diagnosis based on administrative claims data, defined as having an ICD-10-GM diagnosis code for the respective chronic disease recorded with repeated occurrence in at least two distinct treatment cases in 2021 (M2B-criterion^60,61^). For patients with type 1 diabetes, confirmation additionally required at least one documented insulin prescription in 2021. Those enrolled in multiple disease management programs were excluded. The invitation letter contained a link and QR code for digital enrolment through the app- or web-based platform “myoncare” provided by Oncare GmbH. The study comprised four quarterly survey periods over one year. Surveys assessed patient characteristics, generic and disease-specific ePROMs, ePREMs, and health behaviours. At the end of each quarter, respondents received personalized PRO feedback, including individual and peer-group PROM scores, alongside questions evaluating the understandability and perceived usefulness of individual PRO feedback.

### Variables and measures

#### Adherence outcomes—initiation, implementation and persistence

The adherence outcomes for this study were structured around three sequential phases: initiation, implementation, and persistence based on the adherence taxonomy proposed by Vrijens et al.^31^. The first outcome (1) was initiation, defined as a binary component: participant vs. non-participant. Participants were patients who signed up by digitally consenting to the study. The second outcome (2) was implementation, defined as completing at least one ePROM. Prior to ePROM completion participants (initiated patients) were required to complete account registration (processual hurdle), including password creation, and mail verification (steps needed due to German data privacy and medical device registration of the ePROM tool used). Therefore, participants were further categorized as “registrants” (initiated but did not implement) and “respondents” (implemented). The third and last outcome (3) was persistence, measured as the total number of quarterly completed ePROMs (scale 1–4).

#### Patient reported demographic and socioeconomic information

For the initiation analyses (1), demographic data available for both participants and non-participants were used, including disease type, gender, and age group ( <45 years, 45–64 years, 65–74 years, and ≥75 years).

For the implementation (2) and persistence (3) analyses, additional demographic and socioeconomic data were considered, including age, gender, weight, height, education, occupation, household income, and household size. The implementation and persistence analyses controlled for age, gender, disease type, education groups, and household income groups. Due to small sample sizes, monthly household income was categorized as lower (<€2000), middle (€2000–€3499), and higher (≥€3500). Education was categorized as lower (no/secondary school diploma), middle (intermediate school diploma), higher (university entrance qualification or degree), and other educational qualification.

#### Digital questionnaires

The generic PROM, “Patient-Reported Outcomes Measurement Information System - Preference Score” (PROMIS-PROPr)^62^, was used to assess general health outcomes across all four chronic disease areas. PROMIS-PROPr, a validated 14-item questionnaire, is a preference-based score summarizing seven health domains - cognitive function, depression, fatigue, pain, physical function, sleep, and social roles - into a single score. Furthermore, disease-specific PROMs were used to assess disease-specific health outcomes:

- “Asthma Impairment and Risk Questionnaire” (AIRQ) for asthma^63^,
- “Problem Areas In Diabetes” (PAID-5) for diabetes type 1 and 2^64^,
- “Clinical COPD Questionnaire” (CCQ) for COPD^65^,
- “Seattle Angina Questionnaire” (SAQ-7)^66^ and “Rose Dyspnea Scale” (RDS)^67^ for CAD

All instruments were validated in German for use in chronic diseases and were normalized to a scale with values ranging from 0–100, where higher scores indicated better health outcomes.

Alongside these instruments, participants completed additional sets of questions: An 11-item questionnaire assessing the patient’s experiences with the healthcare system, and a health-related behaviour survey that included five questions on physical activity, nutrition, smoking, alcohol consumption, and sleep. Lastly, the survey process also incorporated PRO feedback questions evaluating the understandability, usefulness and emotional impact of the PRO feedback using a 5-point Likert scale. A detailed description of the questionnaire selection process can be found in the PROMchronic study protocol^58^.

### Statistical analysis

A descriptive analysis summarized the patient characteristics of participants and responders. To determine the association of demographics with initiation, six multiple logistic regression models were developed, comparing two link functions (logit and cloglog) and considering main effects as well as interaction effects. Models 1, 2, and 3 applied a logit link function, while models 4, 5, and 6 used a cloglog link function. Interaction terms were included in models 1, 2, 4, and 5, with models 1 and 4 representing the most complex specifications, incorporating all two-way interactions between age group, gender, and disease. Models 2 and 5 included selected interaction terms (age group with gender and disease separately), and models 3 and 6 focused only on the main effects. The models were then assessed using four criteria to evaluate their goodness of fit and identify the model with the best balance between complexity and performance: the Akaike Information Criterion (AIC) values^68^, the deviance explained, the area under the ROC curve (AUC)^69^ and the Brier’s score^70^. Following the identification of the best-performing model, the average marginal effects were calculated to assess the impact of each predictor on initiation in the study.

For implementation and persistence, a hurdle regression model^71^ was developed on participant data. This two-component model was structured with one binary component for implementation and a truncated count component for persistence. The implementation was estimated by segmenting participants into “registrants” (implying they did not overcome the hurdle of account registration) and “responders” (implying they overcame the hurdle of account registration), as defined in the section *Outcomes – Initiation, implementation and persistence*. The continuous age variable was median-centred. A Box-Tidwell test was conducted to test the linearity of the continuous variable (age) and the log odds of the outcome variable (implementation). If violated, an additional quadratic term was added to the regression model. Responders who completed one or more (up to 4) ePROM surveys were included in the (zero truncated) persistence analyses. This model class was chosen to account for overinflation due to the high number of “registrants” (binary component) and dispersion (count component) in those who completed one or more ePROMs. The persistence was modelled using a truncated generalized Poisson model, which parametrizes the variance and, consequently, dispersion explicitly^72^. For both components, different model specifications (implementation: logit, probit; persistence: truncated generalized Poisson with log-link, truncated COM-Poisson with log-link) and variables were evaluated using AIC, Bayesian information criterion (BIC) and root mean square error (RMSE) and chosen accordingly. Multicollinearity was checked using the variance inflation factor (VIF).

Analyses were performed using RStudio v. 2024.12.0 Build 467 with two-sided significance tests (α = 0.05). Categorical variables were summarized as counts and percentages of the overall study population, while continuous variables were presented as means and standard deviations.

## Supplementary information

Supplementary Information File

## Data Availability

The datasets generated and/or analysed during the current study are not publicly available due to the inclusion of primary health data from patients, which is subject to strict regulations under German health data protection laws, but are available from the corresponding author on reasonable request.
