# Supplementary material for: Demographic and socioeconomic determinants of adherence in digital patient-reported outcomes among patients with chronic diseases
Source: NPJ Digit Med. 2025 Aug 1;8:492. doi: 10.1038/s41746-025-01899-2 (PMC12317144; doi:10.1038/s41746-025-01899-2)
Supplement: Supplementary file 1 — Supplementary Information File [file 41746_2025_1899_MOESM1_ESM.pdf]

Supplementary Table 1: Model comparison – predictors of initiation

	Model 1<br>Full-Logit	Model 2<br>Simple-Logit	Model 3<br>Main-Logit	Model 4<br>Full-Cloglog	**Model 5**<br>**Simple-Cloglog**	Model 6<br>Main-Cloglog
Intercept	-3.09 (0.06) ***	-3.09 (0.06) ***	-3.24 (0.05) ***	-3.11 (0.06) ***	**-3.11 (0.06)** ***	-3.26 (0.05) ***
18-44 years	Reference group					
45-64 years	0.08 (0.07)	0.07 (0.07)	0.09 (0.05)	0.08 (0.07)	**0.07 (0.07)**	0.09 (0.05)
65-74 years	-0.50 (0.09) ***	-0.51 (0.09) ***	-0.26 (0.06) ***	-0.49 (0.09) ***	**-0.50 (0.09)** ***	-0.25 (0.06) ***
>=75 years	-1.41 (0.12) ***	-1.42 (0.12) ***	-1.02 (0.06) ***	-1.39 (0.12) ***	**-1.41 (0.11)** ***	-1.01 (0.06) ***
Female	Reference group					
Male	-0.62 (0.10) ***	-0.61 (0.10) ***	0.07 (0.03) *	-0.61 (0.10) ***	**-0.60 (0.10)** ***	0.07 (0.03) *
Asthma	Reference group					
CAD	-0.08 (0.39)	-0.05 (0.39)	-0.23 (0.05) ***	-0.07 (0.39)	**-0.04 (0.38)**	-0.23 (0.04) ***
COPD	-0.35 (0.25)	-0.31 (0.25)	-0.38 (0.04) ***	-0.34 (0.25)	**-0.30 (0.25)**	-0.37 (0.04) ***
Diabetes	0.09 (0.11)	0.09 (0.11)	-0.46 (0.04) ***	0.08 (0.11)	**0.09 (0.10)**	-0.45 (0.04) ***
18-44 years:Male	Reference group					
45-64 years:Male	0.37 (0.11) ***	0.40 (0.11) ***		0.36 (0.11) ***	**0.39 (0.11)** ***	
65-74 years:Male	0.93 (0.12) ***	0.97 (0.11) ***		0.92 (0.12) ***	**0.95 (0.11)** ***	
>=75 years:Male	1.35 (0.13) ***	1.40 (0.13) ***		1.34 (0.13) ***	**1.38 (0.12)** ***	
18-44 years:CAD	Reference group					
45-64 years:CAD	-0.22 (0.40)	-0.23 (0.40)		-0.21 (0.39)	**-0.23 (0.39)**	
65-74 years:CAD	-0.19 (0.40)	-0.20 (0.40)		-0.19 (0.39)	**-0.20 (0.39)**	
>=75 years:CAD	-0.35 (0.41)	-0.36 (0.40)		-0.35 (0.40)	**-0.36 (0.40)**	
18-44 years:COPD	Reference group					
45-64 years:COPD	-0.11 (0.26)	-0.12 (0.26)		-0.11 (0.26)	**-0.11 (0.26)**	
-0.10 (0.26)	-0.11 (0.26)	-0.11 (0.26)		-0.11 (0.26)	**-0.11 (0.26)**	
>=75 years:COPD	-0.01 (0.28)	-0.00 (0.28)		-0.02 (0.28)	**-0.01 (0.28)**	
18-44 years:Diabetes	Reference group					
45-64 years:Diabetes	-0.51 (0.12) ***	-0.52 (0.12) ***		-0.50 (0.12) ***	**-0.51 (0.12)** ***	
65-74 years:Diabetes	-0.64 (0.14) ***	-0.65 (0.14) ***		-0.64 (0.14) ***	**-0.64 (0.14)** ***	
>=75 years:Diabetes	-0.82 (0.17) ***	-0.83 (0.17) ***		-0.82 (0.17) ***	**-0.82 (0.17)** ***	
Male:Asthma	Reference group					
Male:CAD	0.05 (0.10)			0.05 (0.09)		
Male:COPD	0.11 (0.09)			0.11 (0.09)		
Male:Diabetes	0.02 (0.09)			0.02 (0.09)		
AIC	42991.58	42987.18	43193.50	42991.55	**42987.14**	43193.79
BIC	43226.36	43191.33	43275.16	43226.33	**43191.29**	43275.45
Dev.	42945.58	42947.18	43177.50	42945.55	**42947.14**	43177.79
Dev. explained	2.93%	2.92%	2.40%	2.93%	**2.92%**	2.40%
AUC	0.64791	0.64780	0.63626	0.64791	**0.64780**	0.63626
Brier's Score	0.02256	0.02256	0.02259	0.02256	**0.02256**	0.02259
Pseudo R^2	0.03	0.03	0.03	0.03	**0.03**	0.03

Supplementary Table 2: Model comparison – predictors of implementation and persistence

Binary component – implementation

	**Model 1 (logit)**		Model 2 (probit)	
	**β**	**p-value**	β	p-value
Intercept	**1.126**	**<0.001**	0.693	<0.001
Female	**Reference group**			
Male	**-0.055**	**0.474**	-0.033	0.480
Age	**0.015**	**<0.001**	0.009	<0.001
Age-squared	**-0.001**	**<0.001**	-0.000	<0.001
Asthma	**Reference group**			
COPD	**-0.154**	**0.131**	-0.085	0.127
Diabetes	**-0.311**	**0.002**	-0.190	0.002
CAD	**0.021**	**0.849**	0.013	0.852
Higher education	**Reference group**			
Lower education	**-0.179**	**0.072**	-0.110	0.073
Middle education	**-0.002**	**0.985**	-0.002	0.968
Other education	**-0.310**	**0.198**	-0.193	0.197
Higher income	**Reference group**			
Lower income	**-0.268**	**0.019**	-0.160	0.021
Middle income	**-0.221**	**0.031**	-0.221	0.031
N/A income	**-0.549**	**<0.001**	-0.334	<0.001
AIC	**4628.938**		4629.166	
BIC	**4709.445**		4709.673	
Pseudo R^2	**0.034**		0.034	

Count component – persistence

Models	AIC	BIC
**Model A.1**	**6731.428**	**6771.731**
Model A.2	6731.917	6777.976
Model A.3	6734.374	6786.192
Model A.4	6735.928	6793.502
Model A.5	6737.177	6806.267
Model A.6	6744.673	6842.550
Model B.1	6839.223	6879.525
Model B.2	6839.668	6885.728
Model B.3	6842.080	6893.897
Model B.4	6844.174	6901.749
Model B.5	6846.436	6915.525
Model B.6	6854.307	6952.184

Model A: Truncated Generalized Poisson;
Model B: COM-Poisson

Model specifications:
.1 = age + gender + disease
.2 = age + age-squared + gender + disease
.3 = age + gender + disease + education
.4 = age + gender + disease + income
.5 = age + gender + disease + work situation
.6 = age + gender + disease + education + income + work situation
